# ChimerDB 4.0: an updated and expanded database of fusion genes

**DOI:** 10.1093/nar/gkz1013

**Published:** 2019-11-04

**Authors:** Ye Eun Jang, Insu Jang, Sunkyu Kim, Subin Cho, Daehan Kim, Keonwoo Kim, Jaewon Kim, Jimin Hwang, Sangok Kim, Jaesang Kim, Jaewoo Kang, Byungwook Lee, Sanghyuk Lee

**Affiliations:** 1 Department of Bio-Information Science, Ewha Womans University, Seoul 03760, Republic of Korea; 2 Korean Bioinformation Center, Korean Research Institute of Bioscience and Biotechnology, Daejeon 34141, Republic of Korea; 3 Department of Computer Science and Engineering, Korea University, Seoul 02841, Republic of Korea; 4 Department of Life Science, Ewha Womans University, Seoul 03760, Republic of Korea

## Abstract

Fusion genes represent an important class of biomarkers and therapeutic targets in cancer. ChimerDB is a comprehensive database of fusion genes encompassing analysis of deep sequencing data (ChimerSeq) and text mining of publications (ChimerPub) with extensive manual annotations (ChimerKB). In this update, we present all three modules substantially enhanced by incorporating the recent flood of deep sequencing data and related publications. ChimerSeq now covers all 10 565 patients in the TCGA project, with compilation of computational results from two reliable programs of STAR-Fusion and FusionScan with several public resources. In sum, ChimerSeq includes 65 945 fusion candidates, 21 106 of which were predicted by multiple programs (ChimerSeq-Plus). ChimerPub has been upgraded by applying a deep learning method for text mining followed by extensive manual curation, which yielded 1257 fusion genes including 777 cases with experimental supports (ChimerPub-Plus). ChimerKB includes 1597 fusion genes with publication support, experimental evidences and breakpoint information. Importantly, we implemented several new features to aid estimation of functional significance, including the fusion structure viewer with domain information, gene expression plot of fusion positive versus negative patients and a STRING network viewer. The user interface also was greatly enhanced by applying responsive web design. ChimerDB 4.0 is available at http://www.kobic.re.kr/chimerdb/.

## INTRODUCTION

Fusion genes continue to serve as an important source of biomarkers and therapeutic targets in various types of cancer. Since the groundbreaking discovery of *BCR–ABL1* fusion gene in leukemia, numerous driver fusion alterations have been identified as druggable targets, including genes such as *TMPRSS2*, *ALK*, *RET*, *FGFR3*, *ROS1* and *ESR1*, leading to development of targeted therapies (1,2). Additionally, many fusion genes function as biomarkers for specific cancer types as can be seen in the examples of *DNAJB1–PRKACA* fusion in fibrolamella carcinoma (3) and *EWSR1–FLI1* in Ewing's sarcoma (4). Furthermore, a number of fusion genes have been identified as prognostic markers with biological roles. For example, fusion events in metastatic ER-positive breast cancer are more frequent than in primary cases, suggesting fusions as biomarkers of advanced and aggressive disease (5). Thus, fast and reliable identification of fusion genes is increasingly relevant for clinical and pharmaceutical applications.

Since the last update of ChimerDB 3.0 (6), an enormous amount of RNA-Seq data, the major source of mining fusion transcripts, has been released in public. The TCGA dataset represents the largest collection including 13 786 tumor samples in 33 cancer types, which were analyzed by the following two groups independently. Verhaak and colleagues built the TumorFusions database (7) that identified 20 731 gene fusions from 9966 tumor samples and 648 normal specimens in the TCGA database applying their own computational pipeline PRADA (8). The Fusion Analysis Working Group (FAWG) of the TCGA research network investigated 9624 tumors using multiple fusion calling tools and identified 25 664 ‘reliable’ fusion events (9).

Reflecting the importance of fusion genes in cancer, numerous algorithms and databases have been developed to predict and catalog the fusion genes. Li and colleagues carried out comparative performance test for 12 public programs (10). Several more recent programs such as GFusion (11), FusionScan (12) and STAR-Fusion (13) claimed to have achieved higher sensitivity with less false positives. In addition, with so many fusion candidates predicted from transcriptome data, it is also critical to rapidly assess their reliability, functional significance and biological roles. Thus, data aggregation and functional annotation are necessary, ideally with powerful visualization support. INTEGRATE-Vis is a comprehensive visualization tool for gene fusion events (14). FusionGDB provides extensive functional annotations for fusion events aggregated from public resources such as TumorFusions (7), TCGA FAWG (9) and ChiTaRS 3.1 (15). More recently, FusionHub introduced an integrated web platform that supports both annotation and visualization for the largest collection of fusion gene datasets aggregated from 24 resources (16).

The previous version ChimerDB 3.0 was a unique effort to provide comprehensive pictures of fusion genes consisting of several modules with different purposes—ChimerKB as a knowledgebase with extensive manual annotation, ChimerPub as a text-mining utility for identifying fusion genes from PubMed abstracts and ChimerSeq to aggregate the prediction results by analyzing transcriptome sequencing data. To the best of our knowledge, ChimerPub was the first attempt of using text-mining technique to catalog the fusion gene events from the literature. In this update, we describe improvements for each of the three modules and new tools implemented to aid evaluating functional significance of fusion genes. Unlike other resources described earlier, ChimerDB provides a wide range of information encompassing known fusion genes and literature reports as well as candidates from deep sequencing data. ChimerDB would continue to be a comprehensive up-to-date database of fusion genes.

## SYSTEM UPDATE AND METHODS

**Figure 1.**
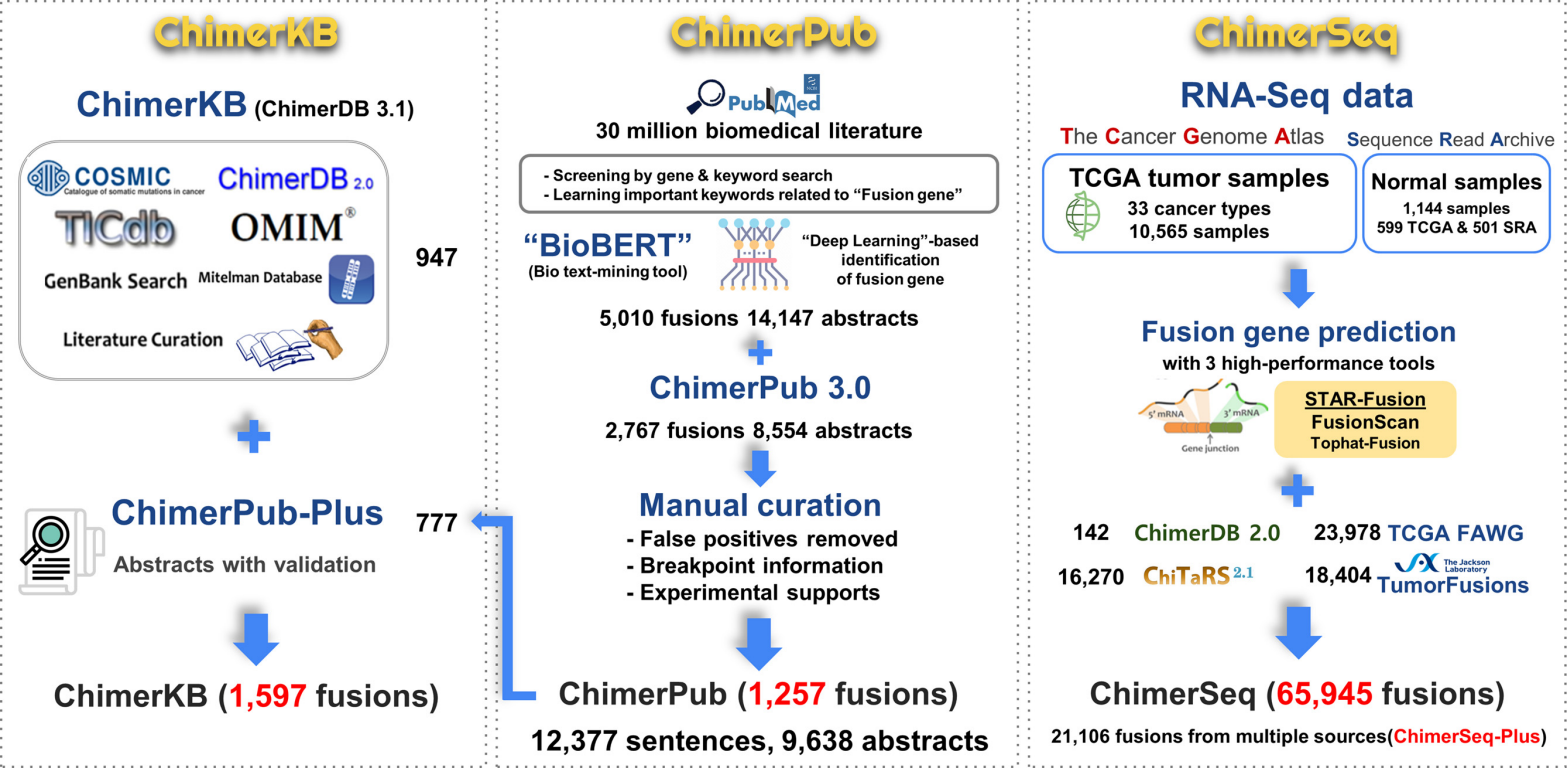
Overview of ChimerDB 4.0. Each number indicates the number of unique gene pairs from the relevant resources.

The overall procedure and contents of ChimerDB 4.0 are summarized in Figure 1. The tripartite module design remains unchanged, but each module was substantially improved. We have developed a new ‘deep learning’-based text-mining method, which was applied to PubMed abstracts to identify 728 new fusion gene entries in ChimerPub update. Importantly, we manually scrutinized the full text of candidate-reporting papers to annotate the fusion breakpoints and experimental evidences as well as to remove false positives. ChimerSeq re-analyzed the whole TCGA transcriptome data using recently developed reliable programs and combined the results with those of TumorFusions and the TCGA FAWG. The number of patients became more than twice the previous version. ChimerKB was updated to reflect new authentic fusion genes identified from ChimerPub update after extensive manual curation. Methods for building ChimerSeq and ChimerPub are described later with further details provided in the Supplementary Information.

### ChimerSeq module

ChimerSeq module analyzed RNA-Seq data available in public and aggregated the results from other databases or computation results. ChimerDB 3.0 covered 4569 tumor samples in 23 cancer types in the TCGA project. In this update, we re-analyzed 10 565 tumor samples across 33 cancer types downloaded from the GDC data portal of NCI. We have also analyzed 1144 normal samples from the TCGA and SRA (E-MTAB-2836, E-GEUV-1, GSE122401) archives to filter out germline fusions.

Our main objective was to reduce the number of false positives, which would lead to unnecessary efforts in the validation procedure. We used two high-performance fusion detection tools to analyze all TCGA RNA-Seq data—FusionScan (12) and STAR-Fusion (13). STAR-Fusion was added because of its high precision and fast computation. We kept the fusion candidates with ≥2 junction reads or with 1 junction read and ≥2 spanning reads. Fusions from the same gene family or from the paralogous genes were removed because of uncertainties in read alignments. We also filtered out fusion genes of germline origin that were observed in the pool of 1144 normal samples. Of note, however, several well-known fusion genes such as TMPRSS2–ERG fusion were identified in a few normal samples from the TCGA cohort. Thus, we rescued such fusion cases present in ChimerKB with literature evidence. The result was merged with two other public resources that analyzed the same dataset (TumorFusions and the TCGA FAWG). Additionally, we integrated the results from TopHat-Fusion prediction (17), EST and mRNA analysis from ChimerDB 2.0 and ChiTaRS 2.1 as included in ChimerDB 3.0. Most TCGA samples were analyzed by five independent programs or pipelines. We collected fusion cases called by multiple programs as the ‘ChimerSeq-Plus’ group representing a reliable list of fusion gene candidates in cancer.

Functional annotation for fusion genes is important in assessing their significance in cancer. We amassed the gene expression and copy number data of the TCGA samples from the UCSC Xena (https://xena.ucsc.edu). Gene lists for functional classes included kinases in the human kinome database (December 2007 update) (18), oncogenes in ONGene (19) and tumor suppressor genes in TSGene 2.0 (20).

### ChimerPub module

ChimerPub was introduced in ChimerDB 3.0 to extract fusion-related sentences from PubMed abstracts semi-automatically. Initial screening of candidate sentences was based on identifying two gene names joined by a delimiter (e.g. BCR–ABL or BCR/ABL). We subsequently built an elaborate classifier model using feature selection and logistic regression methods, which resulted in 2767 fusion genes from 10 580 sentences.

In this update, we built a new ‘deep learning’-based model to identify fusion relations that are not limited to two gene names joined by delimiters in sentences. Thus, the model can extract fusion relations from sentences in natural language forms without any hand-crafted features. Our model was built on BioBERT (https://github.com/dmis-lab/biobert), which is a ‘deep learning’-based language model that showed state-of-the-art performance in representative biomedical text-mining tasks such as named entity recognition, relation extraction and question answering. We stacked one prediction layer on the last layer of the pretrained BioBERT model.

The training data for the prediction layer were obtained from abstracts in ChimerKB 3.0 by extracting sentences with two gene names using BERN (21), a biomedical entity recognition tool. Resulting sentences were classified into positive and negative datasets based on whether the candidate fusion relation existed in ChimerKB or not. To compare the performance of this new tool with the old version, we divided cases into two classes according to the presence of fusion delimiters. We obtained 1295 positive and 5333 negative sentences as the training dataset for the fusion sentences with delimiters, and 379 positive and 96 993 negative sentences for the fusion sentences without delimiters. We also prepared the test datasets by random selection of candidate sentences followed by manual curation (251 positives and 256 negatives for the class with delimiters, and 230 positives and 266 negatives for the class without delimiters).

For sentences with delimiters, both methods achieved excellent performance with high precision of 0.98 and the recall rate of 0.95 in ChimerPub 3.0 and 0.98 in ChimerPub 4.0. For sentences without fusion delimiters, ChimerPub 4.0 achieved the precision of 0.901 and the recall rate of 0.909, which is still excellent although slightly worse than the results for sentences with delimiters. Thus, ChimerPub 4.0 now supports high-performance text mining of PubMed abstracts whether the fusion delimiter symbols are present or not.

The new ‘deep learning’-based model was applied to analyze ∼30 million PubMed abstracts cumulated up to November 2018. Disease terms, validation methods and translocation information were also extracted from the abstracts. We obtained >14 000 abstracts from this new algorithm, and the total number of abstracts became >17 000 after summing abstracts from ChimerPub 3.0.

Text mining inevitably accompanies many false positives; thus, manual curation is essential to increase the credibility and quality of annotations. In building ChimerPub 4.0, we decided to examine the full text, not just the abstract to annotate fusion-related information as well as to remove false positives. Articles reporting cases in ChimerKB 3.0 were excluded from the manual curation and we manually inspected the full text of remaining 2816 articles. During the process of manual curation, we utilized the HGNC gene synonyms from the BioMart service and the cell–cell interaction database of G. Bader Lab (http://baderlab.org/CellCellInteractions).

Finally, we collected the authentic cases as the ‘ChimerPub-Plus’ group where fusion genes were supported with experimental evidences such as Sanger sequencing, reverse transcriptase-polymerase chain reaction (RT-PCR) or fluorescence *in situ* hybridization (FISH). These ChimerPub-Plus cases were added to ChimerKB since they would meet the stringent requirements to be genuine fusion genes.

## RESULTS

**Figure 2.**
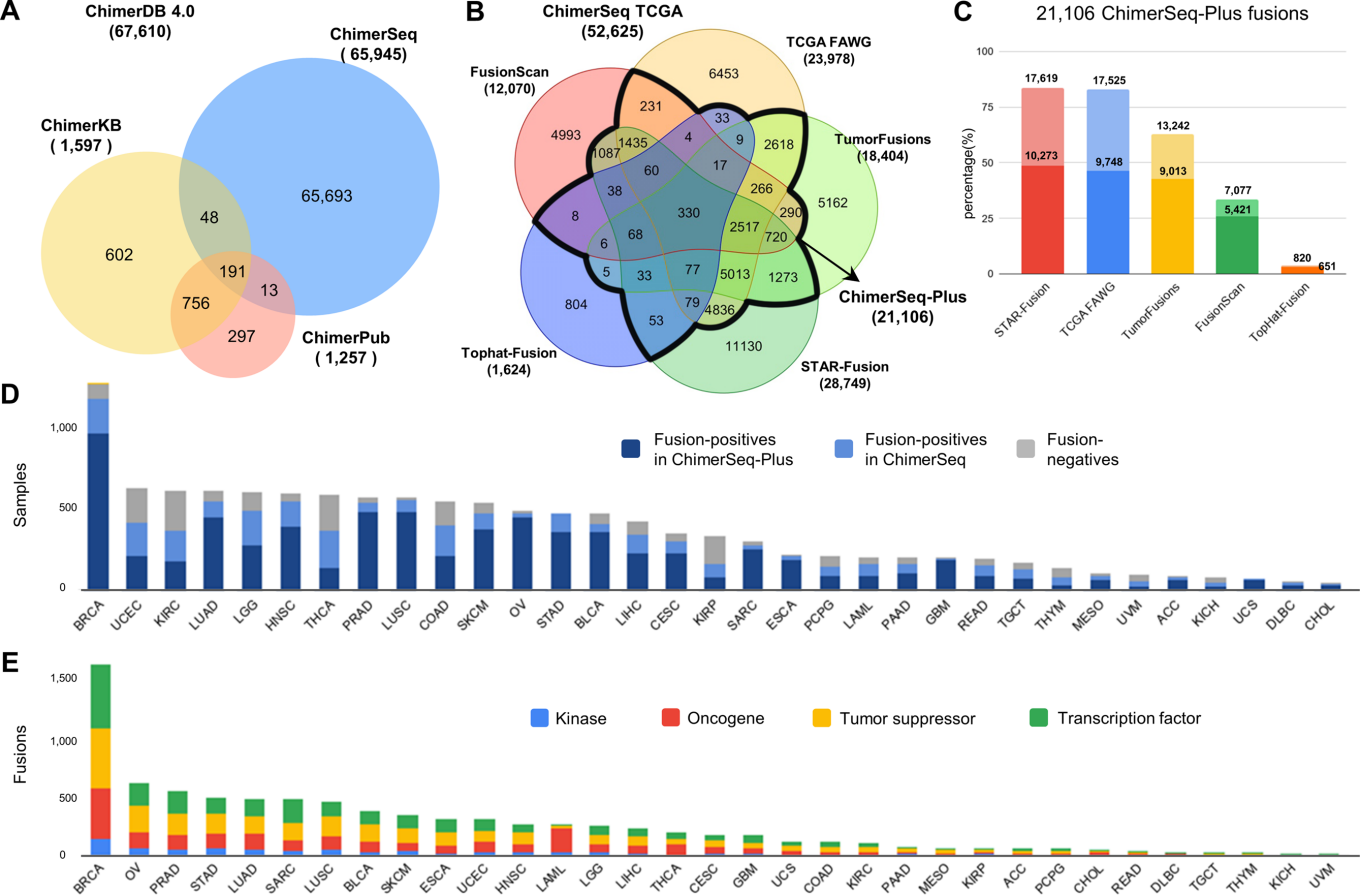
Statistics and contents of ChimerDB 4.0. (**A**) Venn diagram of unique fusions in three modules. (**B**) Venn diagram of unique fusions from five prediction pipelines that analyzed the TCGA dataset. (**C**) Contribution of each prediction pipeline to ChimerSeq-Plus. Dark colors indicate fusion genes that were identified by ≥3 prediction programs, whereas light colors indicate fusion genes predicted by the program of interest and one additional program. (**D**) Bar plot of TCGA samples for each cancer type. (**E**) Bar plot of fusion genes in different functional categories for each cancer type.

ChimerDB 4.0 includes 67 610 fusion gene pairs as summarized in the overall statistics (Table 1). Compared with the previous version (6), the content of ChimerKB and ChimerSeq increased by ∼50% and ∼100%, respectively. ChimerPub's entries, however, decreased from 2767 to 1257 unique fusions due to extensive manual curation with the full-text proof. The overlap among three modules is limited (Figure 2A) implying that three modules play complementary roles.

**Table 1.** Statistics of ChimerDB 4.0

ChimerKB		ChimerPub		ChimerSeq	
Literature curation	147	**Information available**		**TCGA**	49 648
COSMIC	331	Translocation	925	STAR-Fusion	28 749
mRNA Sequence	272	Disease	1075	FusionScan	12 070
Mitelman, OMIM, GenBank	459	Validation method	1049	TumorFusions	18 404
ChimerPub-Plus	777			TCGA FAWG	23 978
				TopHat-Fusion	1624
				**ChimerDB** 2.0	142
				**ChiTaRS** 2.1	16 270
				Panel of **Normals**	2985
**Total**	**1597**	**Total**	**1257**	**Total**	**65 945**
ChimerPub supported	937	ChimerKB supported	937	ChimerKB supported	240
ChimerSeq supported	240	ChimerSeq supported	205	ChimerPub supported	205
				**ChimerSeq-Plus**	21 106
**Known breakpoint cases**				**Novel fusion** ^a^	
Exon junction	1063			TCGA	52 534
				ChiTaRS	16 152

All numbers represent the number of unique fusion genes.

^a^Transcripts not included in ChimerKB and ChimerPub were classified as novel fusion.

ChimerSeq was rebuilt by merging the results from five different pipelines that analyzed the whole TCGA transcriptome data (10 565 samples across 33 cancer types) (Figure 2B, Table 1). TCGA analysis yielded 49 648 fusion genes, representing ∼75% of all fusion genes in ChimerSeq. The proportion of singletons can be an indirect measure of credibility for each prediction method, which increased in the order of TCGA FAWG (27%), TumorFusions (28%), STAR-Fusion (39%), FusionScan (41%) and TopHat-Fusion (50%). Since common predictions from different programs are often regarded as more reliable fusions, we built a new ‘ChimerSeq-Plus’ group that contained 21 106 fusion genes supported by any two of the pipelines. We also examined the contribution of each prediction method to ChimerSeq-Plus, which decreased in the order of STAR-Fusion, TCGA FAWG, TumorFusions, FusionScan and TopHat-Fusion (Figure 2C). However, the portion of fusion genes predicted by ≥3 programs is higher in FusionScan and TopHat-Fusion, implying that their predictions were precise (i.e. low false discovery rate). The number of samples analyzed and the number of fusion genes identified for each cancer type are shown in Figure 2D.

**Figure 3.**
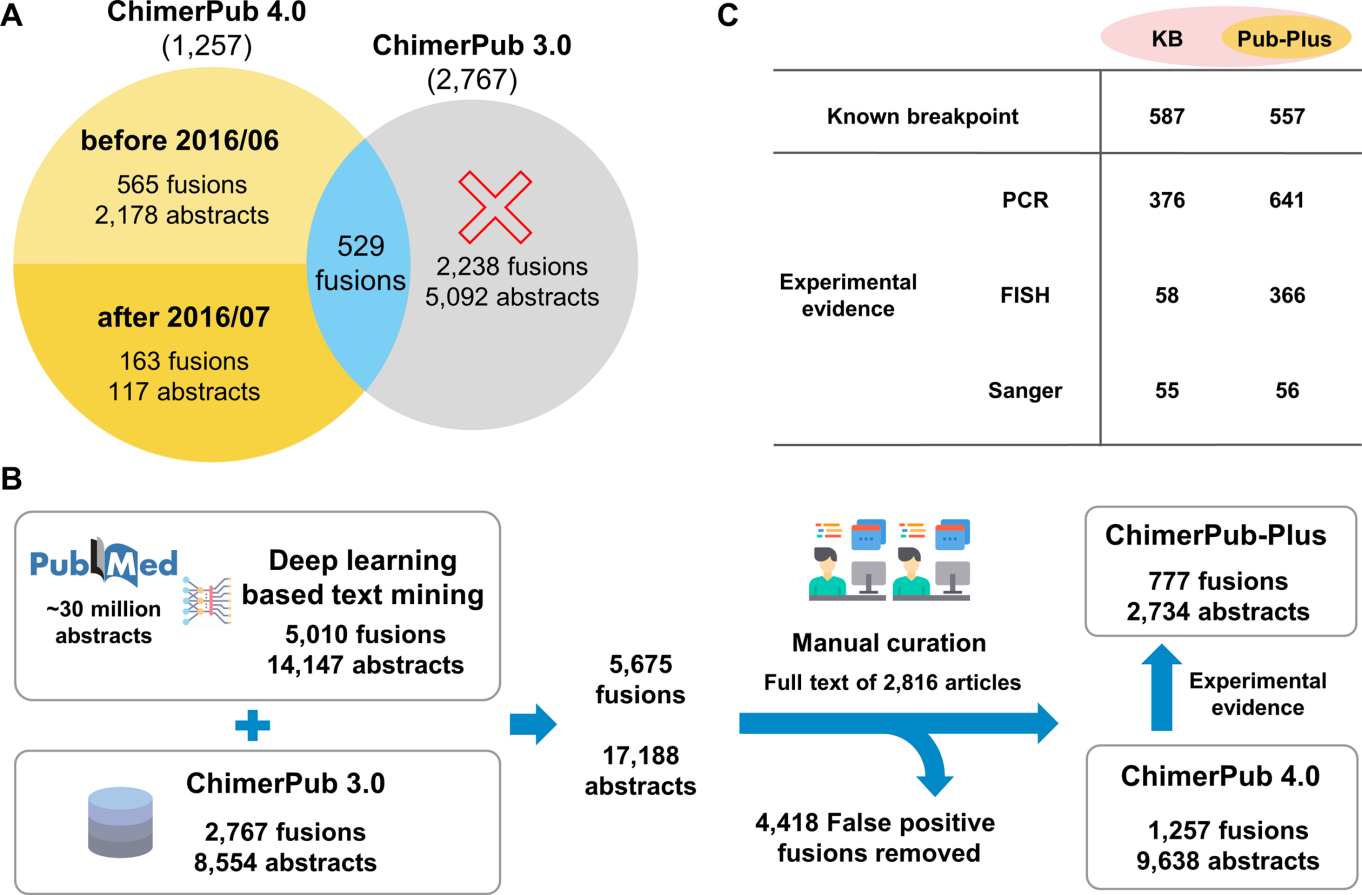
Statistics and contents of ChimerPub 4.0. (**A**) Comparison of ChimerPub 4.0 versus 3.0. (**B**) Curative procedure and resulting numbers at each step. (**C**) Number of ChimerKB entries with information on breakpoints and/or experimental evidences.

ChimerPub was greatly enhanced by implementing a new ‘deep learning’-based algorithm and by extensive manual curation of full-text articles. Our text-mining tool can identify sentences with fusion genes even when two gene are not necessarily joined by a delimiter symbol such as ‘–’ or ‘/’. ChimerPub 4.0 now contains 1257 fusion genes with 728 fusions as new members (Figure 3A). We have 565 fusion genes from articles before June 2016, which should be the contribution from the new deep learning method. This number is comparable to that of the previous symbol-based method (529 fusion genes), illustrating the power of new algorithm. Moreover, a substantial portion of ChimerPub 3.0 (2238 fusion genes) was discarded after thorough curation process as described later.

The manual curation process was extensively reinforced to enhance the quality of fusion records. We obtained 17 188 abstracts (5675 fusion genes) from text mining (Figure 3B). We found that many entries were false positives, where A/B (or A–B) meant the receptor–ligand interactions, gene–gene interactions, signaling or complex relations, or gene synonyms rather than the genuine gene fusion event. Even the gene order was reversed in some cases. The initial round of curation to remove such errors yielded 12 332 articles (2769 fusion genes). Then, articles reporting fusion genes in ChimerKB 3.0 were excluded from the manual curation, leaving 2816 articles (2182 fusion genes) for manual examination with the full-text proof. In the second round of curation, we removed further false positives reporting artificial, synthetic or nonhuman fusions. We have also annotated information on the fusion breakpoints and set of experimental evidence from Sanger sequencing, RT-PCR and FISH.

As a result of manual curation and annotation, a substantial portion of ChimerPub entries became highly reliable with experimental evidences. Thus, we defined the ‘ChimerPub-Plus’ group whose fusion genes were supported by experimental evidences (Figure 3B). We identified 777 such cases and put them into ChimerKB, which greatly expanded the content by ∼50%. The information content of ChimerKB, ChimerPub-Plus is shown in Figure 3C. ChimerKB and ChimerPub now contain 1637 fusion genes with known breakpoints and 1150 fusion genes with experimental supports, which should be the largest collection of this kind.

Fusion genes in ChimerSeq were analyzed for functional roles such as kinases, oncogenes, tumor suppressors or transcription factors across cancer types (Figure 2E). Kinase fusion genes of in-frame are of particular interest because it can alter activity of signaling pathways. We have identified 2629 kinase fusion genes among which 1188 cases are 3′ kinases and 714 cases are in-frame. Likewise, we identified 6706 oncogene-associated, 10 538 tumor suppressor-associated and 9108 transcription factor-associated fusions. Majority of fusions are of CDS–CDS type, thus reading frame change being important to assess their functional significance. Most of known important fusion genes are in-frame fusions because frame shift necessarily leads to loss of function. We found 15 309 (23.2%) fusions as in-frame fusions, 8079 of those belonging to the ChimerSeq-Plus group.

**Figure 4.**
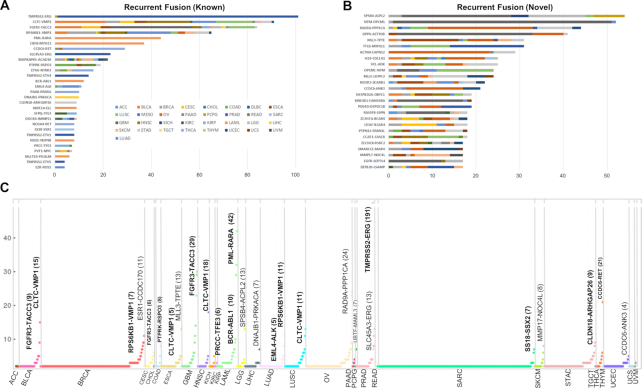
Recurrent fusion genes from the TCGA cohort. (**A**) Representative known fusion genes in ChimerKB and ChimerPub. (**B**) Representative novel fusion genes in ChimerSeq-Plus. (**C**) Recurrent fusion genes for each cancer type. Horizontal axes (**A–C**) indicate the number of patients with fusion genes identified in ChimerSeq-Plus.

Recurrence is the most critical property of clinically important fusion genes. Restricting our attention to fusion genes in ChimerSeq-Plus dataset, we found that 85 fusion genes from ChimerKB or ChimerPub were recurrent (Figure 4A). Additionally, we identified 1293 recurrent fusions where 61 fusions were observed in ≥10 patients (Figure 4B). Of note, novel recurrent fusion genes tend to come from diverse cancer types, highlighting the power of pan-cancer study. Sorting out recurrent fusions according to cancer types illustrates many famous fusion genes such as *TMPRSS2*–*ERG* in PRAD (191 patients), *PML*–*RARA* fusion in LAML (42 patients), *FGFR3*–*TACC3* in GBM (29 patients), *CCDC6*–*RET* in THCA (21 patients), *CLDN18*–*ARHGAP26* in STAD (9 patients) and *EML4*–*ALK* in LUAD (5 patients) (Figure 4C). Thus, mining recurrent genes from ChimerSeq can be an attractive strategy to identify novel cancer biomarkers. The full list of recurrent genes can be downloaded from the website.

## USER INTERFACE

**Figure 5.**
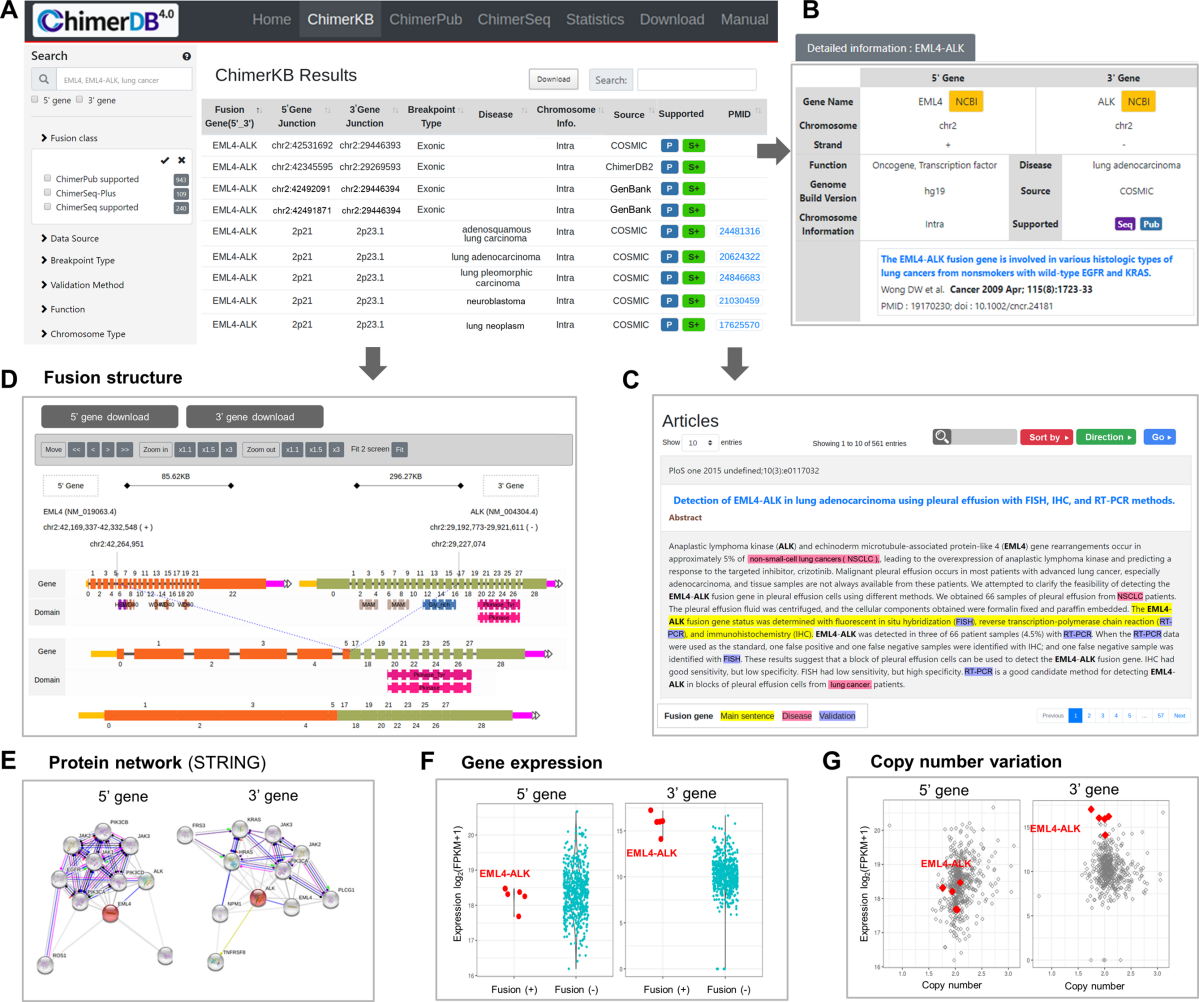
User interface of ChimerDB 4.0. (**A**) The search and filter window and output table in ChimerKB. (**B**) Main output form for a ChimerKB entry. Colored blocks are links to detailed information. (**C**) Example of a PubMed abstract where key words are highlighted. (**D**) Example of fusion structure viewer. (**E**) STRING network view. (**F**) Gene expression plots of 5′ and 3′ genes in fusion-positive versus fusion-negative patients in the TCGA dataset. (**G**) Scatter plots of gene expression versus copy number for 5′ and 3′ genes in the TCGA dataset.

The user interface of ChimerDB adopted a responsive web design, in a similar fashion to the NCI’s GDC data portal. Figure 5 shows the important features in the user interface, taking *EML4–ALK* fusion as an example query. Standard search can be done with gene names or disease terms with the autocomplete function in place (Figure 5A). Users may filter the output list with important features such as data sources, breakpoint information, validation methods, functional classifications, etc. The preloaded numbers and dynamic pie charts allow users to estimate the number of hits in advance. The result is shown in a tabular format where the contents can be searched, sorted and downloaded. The interface also includes links to more detailed information and linkouts to external resources such as NCBI Entrez genes or USCS Genome Browser (Figure 5B). For example, click on a ChimerPub entry opens a new window showing PubMed abstract with important information highlighted (Figure 5C).

In an effort to help users assess functional significance of fusion transcripts of interest, we implemented several novel graphic utilities. Fusion structure viewer shows the transcript structures before and after fusion event, where users can readily view exons, breakpoints and domains (Figure 5D). The graphic supports zoom-in/out and panning for detailed examination. We also added a protein–protein interaction network using the STRING plugin (Figure 5E) (22). Gene expression of 5′ and 3′ genes is informative in assessing the activity of fusion genes. For gene fusion events from the TCGA cohorts, we provide the gene expression plots of 5′ and 3′ genes in the fusion-positive and fusion-negative patients (Figure 5F), such that users can see whether the gene fusion leads to any dysregulation of 5′ or 3′ genes. The scatter plot of gene expression versus copy number provides additional insight into the function of fusion genes (Figure 5G). In the case of *EML4*–*ALK* fusion, it is evident that the fusion event is associated with elevated expression of 3′ *ALK* gene and that this overexpression is independent of copy number variation. Such information would be of great help in sifting candidate fusion genes with functional significance.

## Supplementary Material

gkz1013_Supplemental_File
