# Supplementary material for: ChimerDB 4.0: an updated and expanded database of fusion genes
Source: Nucleic Acids Res. 2019 Nov 4;48(D1):D817–24. doi: 10.1093/nar/gkz1013 (PMC7145594; doi:10.1093/nar/gkz1013)

Supplementary Information for

ChimerDB 4.0: an updated and expanded database of fusion genes

Ye Eun Jang,[1†] Insu Jang,[2†] Sunkyu Kim,[3†] Subin Cho,[1†] Daehan Kim,[3] Keonwoo Kim,[3] Jaewon Kim,[1] Jimin Hwang,[1] Sangok Kim,[4] Jaesang Kim,[4] Jaewoo Kang,[3] Byungwook Lee,[2*] and Sanghyuk Lee[1, 4*]

[1]Department of Bio-Information Science, Ewha Womans University, Seoul 03760, Republic of Korea

[2]Korean Bioinformation Center, Korean Research Institute of Bioscience and Biotechnology, Daejeon 34141, Republic of Korea

[3]Department of Computer Science and Engineering, Korea University, Seoul 02841, Republic of Korea

[4]Department of Life Science, Ewha Womans University, Seoul 03760, Republic of Korea

*Corresponding Authors: Sanghyuk Lee (sanghyuk@ewha.ac.kr) or Byungwook Lee (bulee@kribb.re.kr)

[†] These authors contributed equally to the work.

ChimerSeq Methods

Data sources

All RNA-seq data from the TCGA cohort, including 10,565 tumor samples and 599 normal samples across 33 cancer types, were downloaded from the GDC data portal of NCI. Additional RNA-seq data for 501 normal samples were obtained from the Short Read Archive (SRA) (E-MTAB-2836, E-GEUV-1, and GSE122401).

TumorFusions data were downloaded from the website (https://www.tumorfusions.org, new update in July 2017).

TCGA Fusion Analysis Working Group (FAWG) data were downloaded from the supplementary information of Gao et al. paper (PMID: 29617662).

Programs

STAR-Fusion (Version 1.2.0) was used to analyze all RNA-seq data with the default mode including --annotate, --examine_coding_effect options. GRCh37 human genome was used as the reference genome.

FusionScan (Version 1.0) was used in the default mode using bowtie & ssaha2 mapping tools against the UCSC hg19 (GRCh37) as the reference genome. Output was filtered with the seed(=junction read) ≥ 2.

TopHat-Fusion (Version 0.1.0) was used to build ChimerSeq 3.0. Since TopHat-Fusion took long computation time, we just imported the results of ChimerSeq 3.0 without analyzing additional samples. Thus, TopHat-Fusion result covers only 4,569 tumor samples, not the whole TCGA samples.

We kept the fusion candidates with >2 junction reads or with 1 junction read and >2 spanning reads.

Filtering & Rescuing

Filtering paralogous fusions: Fusions from the same gene family or from the paralogous

genes were removed because of uncertainties in read alignments. We used …

Filtering germline fusions: We filtered out gene fusions identified in the 1,144 normal samples to keep somatic fusions only.

Rescuing known fusions: Since germline filtering removed some of well-known fusions such as TMPRSS2-ERG, we rescued the fusion genes in ChimerKB with literature evidence.

Functional annotations

Gene expression and copy number data of the TCGA samples were downloaded from the UCSC Xena (https://xenabrowser.net). Database version 18.0 (released on July 2019) was downloaded.

Kinase list was obtained from the human kinome for kinases (Dec 2007 update), which included 620 kinase genes (https://kinase.com/human/kinome_download).

Oncogene list was obtained from the ONGene (Dec 26, 2016), which included 803 oncogenes. Liu Y. et al., *J. of Genetics and Genomics* (2017).

Tumor suppressor gene list was obtained from the TSGene 2.0, which included 1,217 genes (Zhao M. et al., *Nucleic Acids Res.* 2016).

Transcription factor gene list was obtained from the Cell review paper on the human transcription factors version 1.01, which included 1,639 TF genes (Lambert SA et al., *Cell* 2018).

Cell-cell interaction database from G. Bader lab was used for the list of receptor genes, ligands, cell-cell interactions (https://baderlab.org/CellCellInteractions).

ChimerPub Methods

Deep learning based model construction

Our fusion gene extraction model was built on BioBERT [1] which is a language model that had obtained state-of-the art performance in representative biomedical text mining tasks such as named entity recognition, relation extraction, and question answering. BioBERT is a contextualized embedding model of biomedical words, which means BioBERT can provide different embedding vectors for the same word depending on the context of the sentence where the word occurs.

Fusion gene relation extraction can be regarded as a sentence classification if two genes are represented as target genes in the sentence [2]. The relation extraction module of BioBERT classifies a sentence using a single fully connected layer based on the embedding vector of the last token of the sentence obtained by BioBERT. When the module classifies a sentence as "True" label, the result means there is a fusion relationship between the two target genes represented in the sentence. To explicitly represent the two target genes in sentence, we replaced the two genes with pre-defined tags (e.g @GeneA$ and @GeneB$). The pre-defined tags are considered a unique word and the embedding vectors of the tags are also fine-tuned when training the model. Since we anonymized the target gene name, the fusion gene extraction model shows consistent performance on other dataset where genes do not occur in training dataset. The source code of the relation extraction module of BioBERT is available at https://github.com/dmis-lab/biobert.

[1] Jinhyuk Lee, Wonjin Yoon, Sungdong Kim, Donghyeon Kim, Sunkyu Kim, Chan Ho So, Jaewoo Kang. BioBERT: a pre-trained biomedical language representation model for biomedical text mining. arXiv preprint arXiv:1901.08746, 2019

[2] Bhasuran, Balu, and Jeyakumar Natarajan. "Automatic extraction of gene-disease associations from literature using joint ensemble learning." *PloS one* 13.7 (2018): e0200699.

Supplementary Figure 1. ChimerPub text mining for extracting abstracts from PubMed

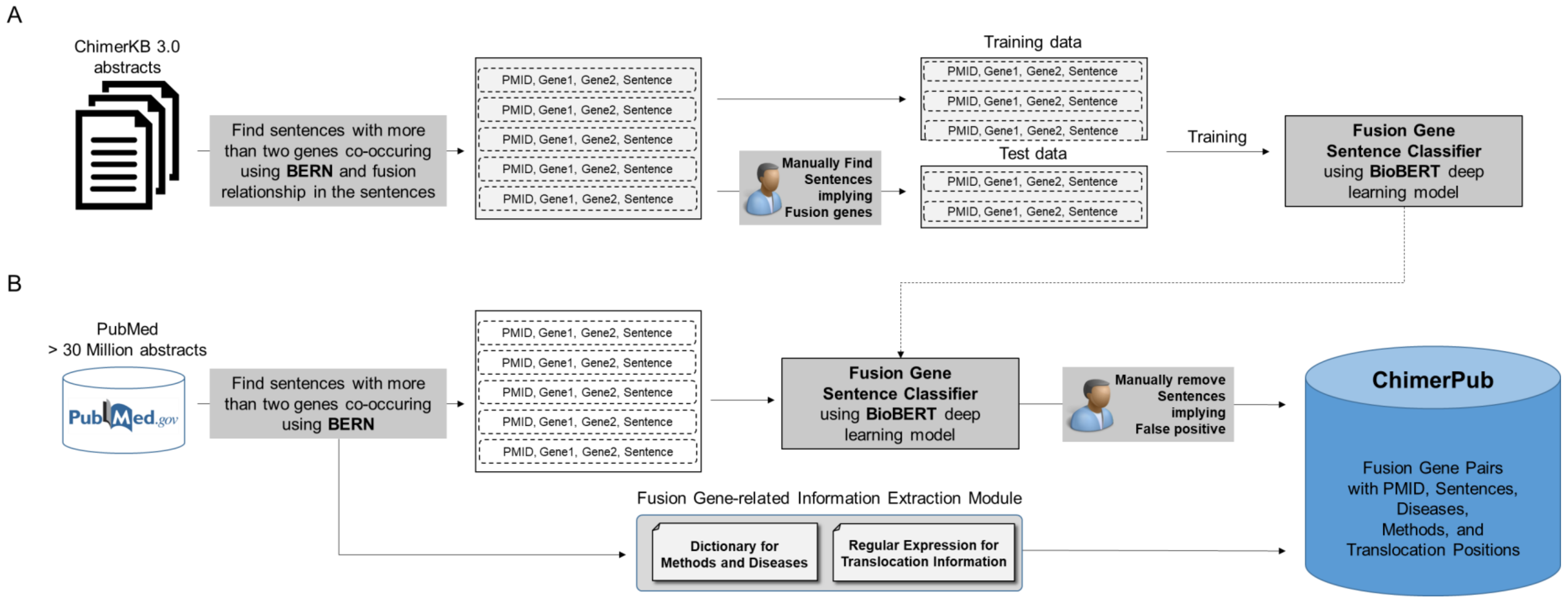

A) Constructing our fusion gene extraction model. We collect all sentences containing two genes in ChimerKB 3.0 abstracts. If two genes in a sentence are listed in ChimerKB, we consider the sentence is positive. We split the sentence data into training and test data, and manually curate the test dataset to evaluate our deep learning model accurately. Then we train our fusion gene extraction model based on BioBERT with the training data and evaluated our model with the test data. **B) Constructing ChimerPub using our fusion gene extraction model.** We collect all sentences containing two genes in the entire PubMed abstracts. Using our trained fusion gene extraction model we infer the labels of the collected sentences and extract sentences containing fusion genes. For the better precision, we manually remove false-positive sentences. the In addition, we extract diseases, experimental methods, translocations and positions about fusion genes from the PubMed abstracts. All the extracted fusion genes and related information are organized and provided in ChimerPub.

Procedure for building ChimerPub 4.0

The overall procedure for building ChimerPub 4.0 is shown in Supplementary Figure 2.

Our text-mining tool was applied to analyzed ~30 million abstracts in PubMed cumulated up to November 2018. We obtained 14,147 abstracts (5,010 fusions) from the new "deep learning"-based algorithm. Combining 8,554 abstracts from ChimerPub 3.0, a total of 17,188 abstracts (5,675 fusions) became the dataset for manual curation.

Initial round of manual curation removed false positives semi-automatically. We removed the fusion candidates where the two genes were related by false information such as receptor-ligand interactions, gene-gene interactions, signaling pathways, complex formation, gene synonyms (aliases). We also corrected the wrong gene order. This process eliminated 4,856 abstracts (2,906 fusions), 1,896 fusions of which were originated from ChimerPub 3.0.

Remaining 12,332 abstracts are still too many for full text curation. So we focused on finding novel fusion genes by keeping aside the articles reporting known fusions in ChimerKB 3.0. We further removed the abstracts whose text-mining score < 0.2, leaving 2,816 articles for full text proof reading.

The second round of manual curation involved further removal of false positives such as artificial or nonhuman fusions. We also looked for annotation information that included the breakpoints and experimental supports such as Sanger sequencing, RT-PCR, and FISH.

Abstracts reporting known fusion genes in ChimerKB 3.0 that were kept aside before full text proof were remerged into ChimerPub 4.0. The final ChimerPub 4.0 included 1,257 fusions from 9,638 abstracts.

Supplementary Figure 2. ChimerPub building with manual curation process.

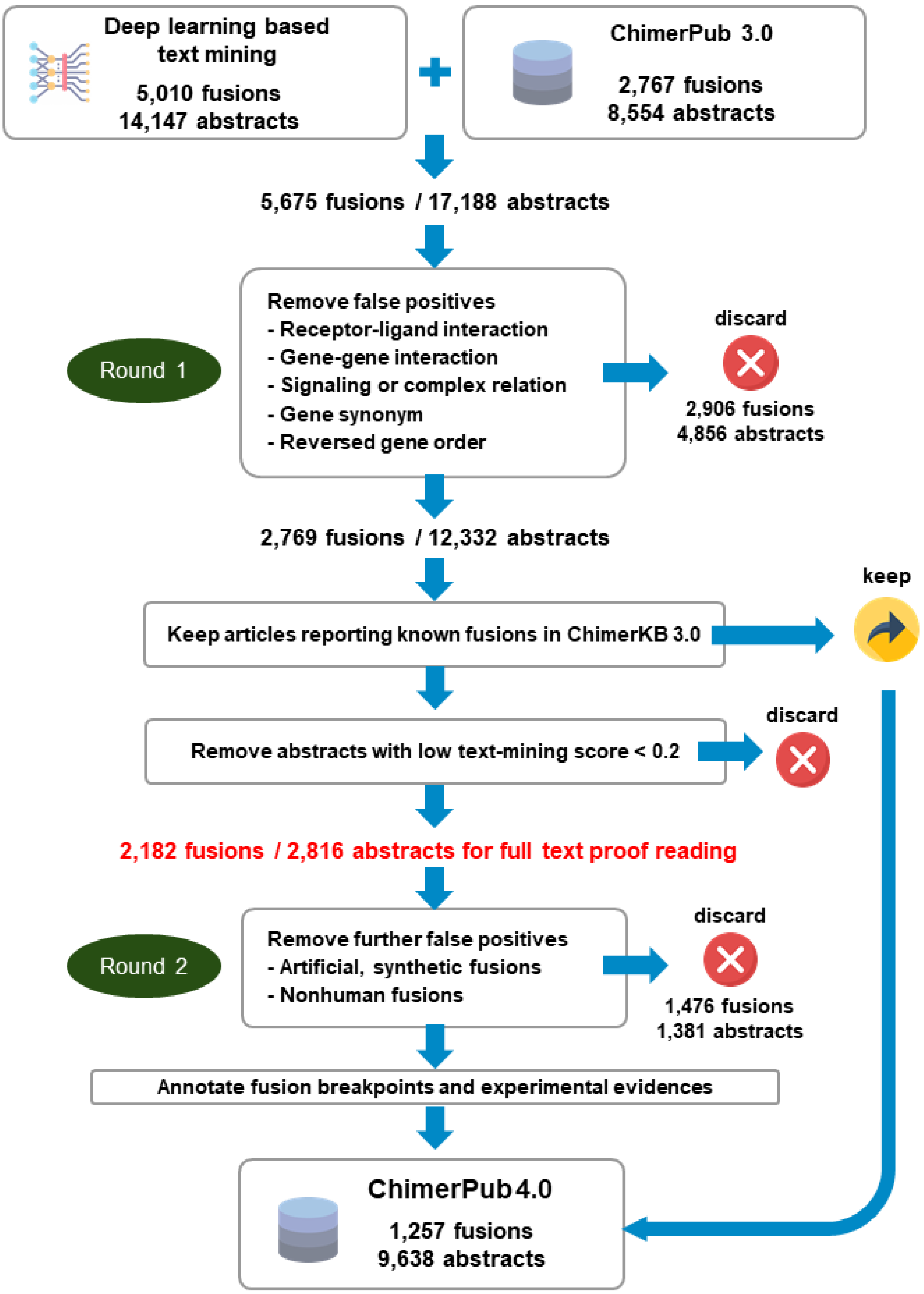

Supplement: gkz1013_Supplemental_File [file gkz1013_supplemental_file.pdf]
